# Evaluation of Rapid Influenza Diagnostic Tests for the Detection of H5N1 in Milk

**DOI:** 10.3390/pathogens14040325

**Published:** 2025-03-28

**Authors:** Missiani Ochwoto, Franziska Kaiser, Claude Kwe Yinda, Arthur Wickenhagen, Vincent J. Munster

**Affiliations:** Laboratory of Virology, Division of Intramural Research, National Institute of Allergy and Infectious Diseases (NIAID), National Institutes of Health (NIH), 903 South 4th Street, Hamilton, MT 59840, USA

**Keywords:** HPAI H5N1, rapid influenza diagnostic test, RIDT, raw milk

## Abstract

Rapid influenza diagnostic tests (RIDTs) could be useful in the current bovine H5N1 outbreak. Here, we evaluated three RIDTs with H5N1. The RDITs showed comparable sensitivity with H5N1 compared to seasonal influenza A virus H3N2, and no difference was observed in sensitivity between raw milk and the PBS control.

## 1. Introduction

Since March 2024, high pathogenic avian influenza (HPAI) A (H5N1) clade 2.3.4.4b has been detected in numerous dairy cattle herds in several states in the United States [1]. Similarly, high titers of this infectious virus have been detected in poultry, other terrestrial mammals, and the milk of infected dairy cattle [2]. The continuous circulation of this novel bovine H5N1 in dairy cattle has raised concerns about potential spillover into other livestock species and pets. In addition, since the start of the outbreak, a total of 66 human cases [3,4] and one death [5] have been confirmed in the United States. The infected patients presented with fever, conjunctivitis, and mild respiratory symptoms.

The ongoing and expanding outbreak of H5N1 in dairy cattle has exposed major gaps in the ability to rapidly perform testing both in the agricultural and public health settings. For influenza A and B viruses, several rapid influenza diagnostic tests (RIDTs) detecting viral nucleoprotein (NP) antigens are commercially available [6,7,8]. Given the high degree of amino acid identity between the NP of all influenza A viruses (IAVs), we evaluated the ability of commercial RIDTs to detect the H5N1 virus in raw cow milk.

## 2. Materials and Methods

We used three commercial RIDTs, namely Fisher Healthcare™ Sure-Vue™ Signature Influenza A and B Test Kit (Pittsburgh, PA, USA), SEKISUI Diagnostics™ OSOM™ Ultra Plus Flu A and B Test Kit, (San Diego, CA, USA) and BioSign^®^ Flu A&B (Princeton BioMedtech Corporation, Monmonth Junction, NJ, USA) to evaluate the ability to detect H5N1 in point-of-care settings [6].

To achieve this, we obtained raw unpasteurized cow’s milk from a local dairy cattle herd that is mostly composed of Jersey dairy cows. The raw milk was transported to the lab and utilized the same day or stored at 4 °C for a maximum of 4 days. A sample of the milk was sent to CentralStar laboratories, WI, for a component analysis.

We used three H5N1 virus isolates—A/Vietnam/1203/2004 H5N1, A/mountain lion/Montana/1/2024 H5N1, and A/bovine/Ohio/B24OSU-342/2024 H5N1—and one H3N2 virus isolate (A/New York/470/2004 H3N2). The virus stocks were standardized and then diluted in either phosphate-buffered saline (PBS) or raw unpasteurized cow’s milk. Ten-fold serial dilutions from 10^7^ to 10^1^ 50% tissue culture infectious doses (TCID_50_) per milliliter were prepared and used in the study. Virus dilutions were performed in triplicate and 50 µL of each dilution was used to perform each RIDT assay according to the manufacturers’ instructions. In addition, we extracted RNA of the dilutions evaluated by the RIDTs using the Qiagen QIAamp Viral RNA Kit, (Qiagen Sciences, Germantown, MD, USA) and copies of genomic RNA targeting the M gene of Influenza A virus were determined by qRT-PCR using TaqMan™ Fast Virus One-Step Master Mix and QuantStudio 6 Flex Real-Time PCR System [9].

We further ran the lowest positive RIDT input on an influenza A nucleoprotein ELISA (Cell Biolabs Inc., San Diego, CA, USA) to determine the amount of nucleoprotein recognized by the RIDTs. The experiments were performed according to the manufacturer’s product manual: briefly, 225 µL of each sample was transferred to a microcentrifuge tube containing 25 µL of 10X lysis buffer. After inactivation at 56 °C for 30 min, 100 µL was added to an Anti-influenza A Nucleoprotein Antibody-coated plate. In the end, the absorbance of each microwell was read on an Agilent BioTek synergy HTX spectrophotometer (Santa Clara, CA, USA), using 450 nm as the primary wavelength.

To test the specificity of the RIDTs, we used three mastitis-causing agents to prepare 1.0 × 10^8^ bacterial colony-forming units in raw unpasteurized cow’s milk. We used 50 µL of each bacterium; *Klebsiella pneumoniae* (ATCC 43816), *Escherichia coli*, (DH10B Cells) and *staphylococcus aureus* (*ATCC 25923*) to perform each RIDT assay according to the manufacturers’ instructions. In addition, we incubated the infected milk at 37 °C overnight while shaking and tested the milk after 24 h.

## 3. Results

The three RIDTs detected the H5N1 virus isolates diluted in either PBS or raw milk, with BioSign^®^ Flu A and B detecting 1log_10_ higher compared to the other RIDT that detected 5000 TCID_50_ (Table 1). For the bovine isolate, we observed a minimally reduced detection with 2/3 (67%) RIDTs being positive when diluted in milk versus 3/3 (100%) RIDTs positive when diluted in PBS. The lower limit of positivity for the H5N1-Mountain lion and H5N1-Bovine isolates was 5000 TCID_50_ on two RIDTs (OSOM and Sure-Vue) and 50,000 TCID_50_ on RIDT (BioSign), which was the same as for the seasonal H3N2 isolate tested. The only exception was the H5N1-Vietnam isolate, which had a lower limit of detection for all three RIDTs at 50,000 TCID_50_.

**Figure 1:**
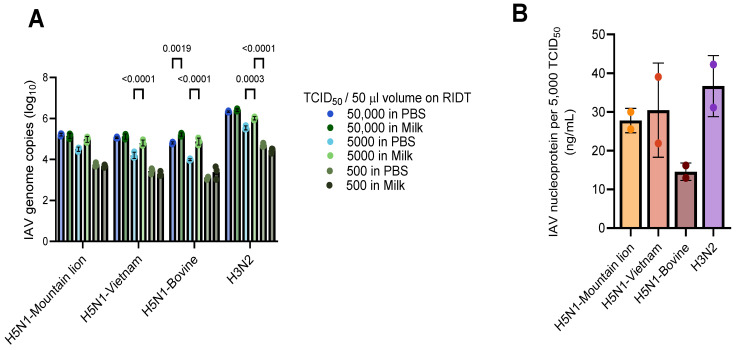
Quantification of rapid influenza diagnostic test (RIDT) input material. (**A**) Number of influenza A virus genome copies per 50 μL volume H5N1 or H3N2 virus isolates diluted to 50,000, 5000, and 500 TCID_50_ in PBS or milk. Each dilution was subsequently tested on each RIDT reported in Table 1. Data are plotted as means ± S.D. of *n* = 3 biological replicates. Statistical analysis was conducted using two-way ANOVA followed by Tukey’s post-test to compare PBS with milk samples per TCID_50_ input. *p*-values < 0.05 are shown. (**B**) Influenza A nucleoprotein ELISA determining amount of IAV nucleoprotein per 5000 TCID_50_ input material for each H5N1 or H3N2 virus isolate tested on RIDTs. Data are plotted as means ± S.D. of *n* = 2 biological replicates. Statistical analysis was conducted using Kruskal–Wallis test followed by Dunn’s multiple comparisons correction. *p*-values < 0.05 are shown.

We determined the amount of IAV genome copies for the two lowest positive RIDT inputs (50,000 and 5000 TCID_50_) to assess whether the dilutions resulted in equal amounts of virus input. While the H5N1-Vietnam and H5N1-Bovine isolates had slight differences in the amount of IAV genome copies present between 5000 TCID_50_ in PBS and milk, the virus input per RIDT was within 1log_10_ genome copies (Figure 1A). The amount of H3N2 dilutions in PBS was slightly higher compared to milk samples, but virus input was <0.5log_10_ genome copies (Figure 1A), indicating similar virus inputs on each RIDT. Next, we determined the amount of IAV nucleoprotein detected within the input of the lowest positive RIDT (5000 TCID_50_), showing a range from 14.6 to 36.7 ng/mL (Figure 1B), further indicating a similar range of virus input on each RIDT.

We next evaluated the potential for false-positive results with the RIDTs using causal bacterial agents of mastitis. All the RITDs tested negative for *Klebsiella pneumoniae*, *Escherichia coli*, and *Staphylococcus aureus*. Similar results were observed when the infected milk was incubated overnight before testing.

When we sent the milk in for a component analysis (CentralStar laboratories, Kaukauna, WI, USA) we obtained a fat content of 4.98% and a 3.51% protein content, which was within a typical range for Jersey cows. We next did a 4-fold dilution of milk in PBS to assess the effect of the raw milk matrices on the performance of the RITDs. We did not observe any significant differences when we diluted the milk in PBS and virus concentrations 5000 TCID_50_ and 500 TCID_50_. The lowest positive test was at 5000 TCID_50_ for all the RITDs regardless of milk/PBS concentration. While performing the RITD testing, we did not observe any notable changes that could affect the quality of the results such as sample clogging. In addition, the RITD performance was the same when performed with milk at 4 °C compared to milk at room temperature. For all the tests, the time taken to read the result was as described by the manufacturer.

## 4. Discussion

The circulation of HPAI H5N1 in dairy cattle and the risk for zoonotic and cross-species transmission [5,10] highlights the need for easy and readily implementable diagnostic assays. In this study, the three evaluated RIDTs detected various strains of HPAI H5N1 with similar sensitivity as seasonal H3N2. This suggests that these assays can be used to detect HPAI H5N1 in samples obtained from infected animals, for instance, such as cats.

In addition, HPAI H5N1 spiked in raw milk can be detected with the same sensitivity as the virus diluted in PBS, by all tested RIDTs. We therefore demonstrate that these RIDTs can be used to screen HPAI H5N1 in raw milk samples of suspected dairy cattle and provide a rapid on-site assessment of the infection status of the cattle herd. Testing on site is economical and RIDTs are cost-effective compared to molecular diagnostic rests [11]; therefore, the test can be narrowed down to which cow among the herd is infected. This will prevent and control infection within a herd, making it easier to be used at a point of care. Similarly, the RIDTs can be used, just like SARS-CoV-2 preliminary or emergency medical screening assays [12], to diagnose populations at risk of contracting HPAI H5N1. The RITD kits were specific, and contaminants like causative agents for mastitis in milk will not affect the results. The major limitation of RDITs is the inability to differentiate between influenza A virus subtypes (e.g., H1, H3, and H5), and positive samples should undergo differential molecular diagnostics for further characterization and subtyping. The limitation of this study was that the study utilized spiked raw milk rather than milk from infected cows; therefore, RIDTS should next be evaluated in a field setting.

## 5. Conclusions

In conclusion, RIDTs could be valuable and cost-effective tools for continued and increased HPAI H5N1 surveillance.

## Figures and Tables

**Table 1:** Evaluation of the performance of rapid influenza diagnostic tests (RIDTs) for influenza A isolates diluted in either phosphate-buffered saline (PBS) or raw unpasteurized cow’s milk.

Influenza A Virus Isolate	RIDT	Virus Concentration on RIDT (TCID_50_)					
		50,000	5000	500	50,000	5000	500
		Diluted in Raw Cow’s Milk			Diluted in PBS		
H3N2 A/New York/470/2004	OSOM	3/3 (100%)	3/3 (100%)	0/3 (0%)	3/3 (100%)	3/3 (100%)	0/3 (0%)
	Sure-Vue	3/3 (100%)	3/3 (100%)	0/3 (0%)	3/3 (100%)	3/3 (100%)	0/3 (0%)
	BioSign	3/3 (100%)	0/3 (0%)	0/3 (0%)	3/3 (100%)	0/3 (0%)	0/3 (0%)
H5N1 A/mountain lion/Montana/1/2024	OSOM	3/3(100%)	3/3 (100%)	0/3 (0%)	3/3 (100%)	3/3 (100%)	0/3 (0%)
	Sure-Vue	3/3 (100%)	3/3 (100%)	0/3 (0%)	3/3 (100%)	3/3 (100%)	0/3 (0%)
	BioSign	3/3 (100%)	0/3 (0%)	0/3 (0%)	3/3 (100%)	0/3 (0%)	0/3 (0%)
H5N1 A/Vietnam/1203/2004	OSOM	3/3 (100%)	0/3 (0%)	0/3 (0%)	3/3 (100%)	0/3 (0%)	0/3 (0%)
	Sure-Vue	3/3 (100%)	0/3 (0%)	0/3 (0%)	3/3 (100%)	0/3 (0%)	0/3 (0%)
	BioSign	3/3 (100%)	0/3 (0%)	0/3 (0%)	3/3 (100%)	0/3 (0%)	0/3 (0%)
H5N1 A/bovine/Ohio/B24OSU-342/2024	OSOM	3/3 (100%)	2/3 (67%)	0/3 (0%)	3/3 (100%)	3/3 (100%)	0/3 (0%)
	Sure-Vue	3/3 (100%)	2/3 (67%)	0/3 (0%)	3/3 (100%)	3/3 (100%)	0/3 (0%)
	BioSign	3/3 (100%)	0/3 (0%)	0/3 (0%)	3/3 (100%)	0/3 (0%)	0/3 (0%)

## Data Availability

https://figshare.com/s/356321e80c33a400c011 (accessed on 25 March 2025).

## Abbreviations

The following abbreviations are used in this manuscript:

HPAI H5N1	Highly pathogenic avian influenza (HPAI) A (H5N1)
IAV	Influenza A virus
NP	Nucleoprotein
PBS	Phosphate-buffered saline
RIDTs	Rapid influenza diagnostic tests
S.D.	Standard deviation
SARS-CoV-2	Severe acute respiratory syndrome Coronavirus 2
TCID_50_	50% tissue culture infectious doses
